# Supplementary material for: Complex‐centric proteome profiling by SEC‐SWATH‐MS
Source: Mol Syst Biol. 2019 Jan 14;15(1):e8438. doi: 10.15252/msb.20188438 (PMC6346213; doi:10.15252/msb.20188438)
Supplement: Supplementary file 8 — Dataset EV7 [file MSB-15-e8438-s008.zip › feature_plots_string/P59780.pdf]

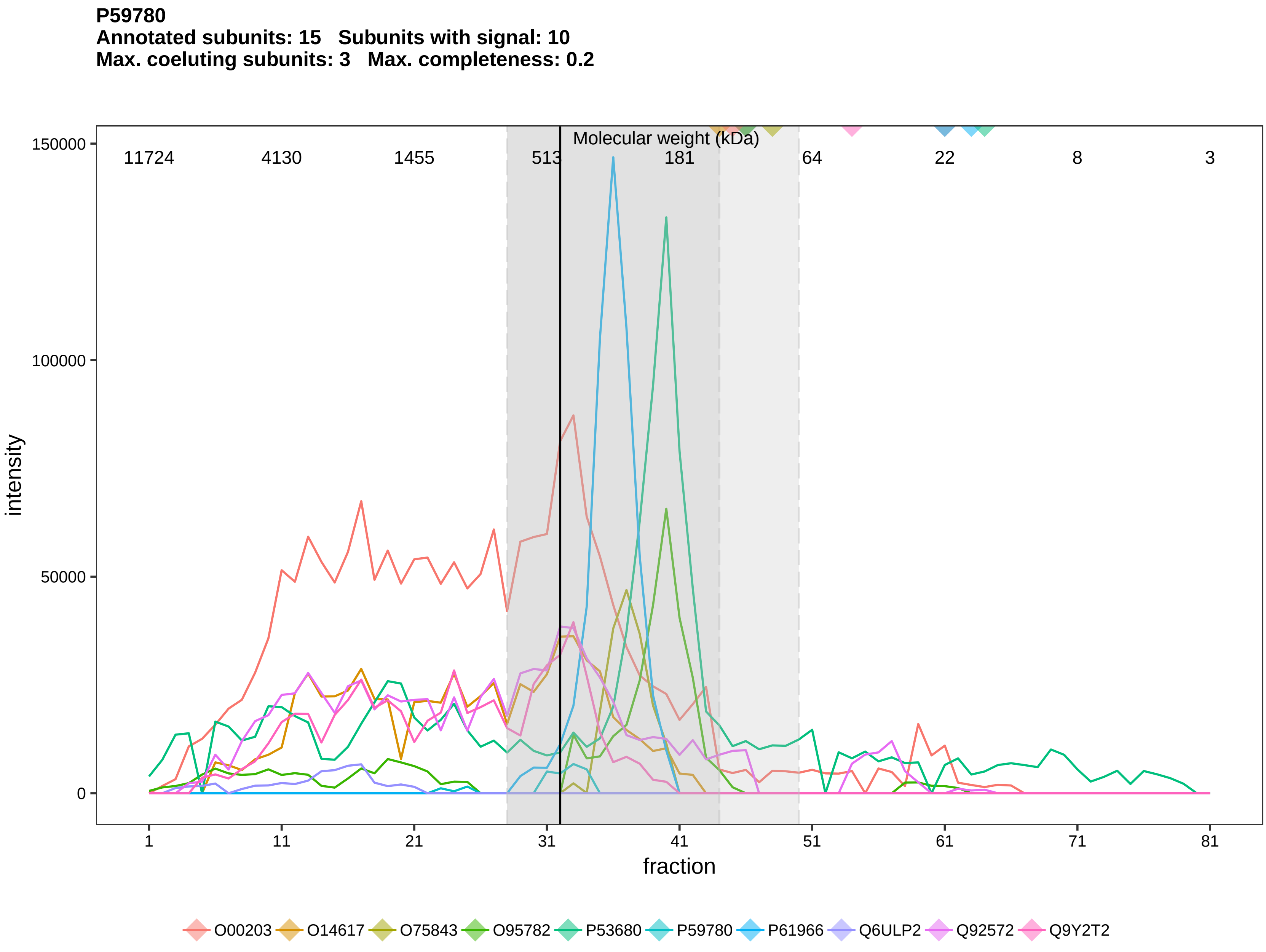
P59780
Annotated subunits: 15 Subunits with signal: 10
Max. coeluting subunits: 3 Max. completeness: 0.2
Molecular weight (kDa)
11724
4130
1455
513
181
64
22
8
3
150000
100000
50000
0
intensity
1
11
21
31
41
51
61
71
81
fraction
O00203
O14617
O75843
O95782
P53680
P59780
P61966
Q6ULP2
Q92572
Q9Y2T2
